# Supplementary material for: Effect of bosentan in pulmonary hypertension development in systemic sclerosis patients with digital ulcers
Source: PLoS One. 2020 Dec 10;15(12):e0243651. doi: 10.1371/journal.pone.0243651 (PMC7728198; doi:10.1371/journal.pone.0243651)
Supplement: Attachment — Submitted filename: Response to reviewers.docx [file pone.0243651.s002.docx]

**Response to Editor and Reviewers**

Dear Dr. Navarini, I hope you are well. First of all I would like to thank you and the reviewers your comments about our manuscript. We have read all the considerations with high interest. We are happy to notify that we modified the manuscript and answered your suggestions in the next lines. Please don't hesitate to contact with us if you need more requirements.

All the best

Ivan Castellví, MD; PhD.

**Response to Editor:**

**Journal Requirements:**

**When submitting your revision, we need you to address these additional requirements.**

**1. Please ensure that your manuscript meets PLOS ONE's style requirements, including those for file naming. The PLOS ONE style templates can be found at**

**https://journals.plos.org/plosone/s/file?id=wjVg/PLOSOne_formatting_sample_main_body.pdf and**

**https://journals.plos.org/plosone/s/file?id=ba62/PLOSOne_formatting_sample_title_authors_affiliations.pdf**

1. We reviewed all the manuscript to meet PLOS ONE’s style requirements.

**2. Please refer to any post-hoc corrections to correct for multiple comparisons during your statistical analyses. If these were not performed please justify the reasons. Please refer to our statistical reporting guidelines for assistance (https://journals.plos.org/plosone/s/submission-guidelines.#loc-statistical-reporting).**

2. Done. Taking into consideration our design A post-hoc analysis was not performed. We included those explanation in material and methods

**3. Please also clarify whether the data you used for this study was de-identified or anonymized before you had access to it.**

3. We clarified it in Material and Methods part of the manuscript. Data of the patients were encrypted before to start our retrospective study.

**4. In your Data Availability statement, you have not specified where the minimal data set underlying the results described in your manuscript can be found. PLOS defines a study's minimal data set as the underlying data used to reach the conclusions drawn in the manuscript and any additional data required to replicate the reported study findings in their entirety. All PLOS journals require that the minimal data set be made fully available. For more information about our data policy, please see http://journals.plos.org/plosone/s/data-availability.**

**Upon re-submitting your revised manuscript, please upload your study’s minimal underlying data set as either Supporting Information files or to a stable, public repository and include the relevant URLs, DOIs, or accession numbers within your revised cover letter. For a list of acceptable repositories, please see http://journals.plos.org/plosone/s/data-availability#loc-recommended-repositories. Any potentially identifying patient information must be fully anonymized.**

**Important: If there are ethical or legal restrictions to sharing your data publicly, please explain these restrictions in detail. Please see our guidelines for more information on what we consider unacceptable restrictions to publicly sharing data: http://journals.plos.org/plosone/s/data-availability#loc-unacceptable-data-access-restrictions. Note that it is not acceptable for the authors to be the sole named individuals responsible for ensuring data access.**

**We will update your Data Availability statement to reflect the information you provide in your cover letter.**

4. Thank you for your reminder. We will upload our minimal data set as a Supporting Information file.

**5. We note that you have included the phrase “data not shown” in your manuscript. Unfortunately, this does not meet our data sharing requirements. PLOS does not permit references to inaccessible data. We require that authors provide all relevant data within the paper, Supporting Information files, or in an acceptable, public repository. Please add a citation to support this phrase or upload the data that corresponds with these findings to a stable repository (such as Figshare or Dryad) and provide and URLs, DOIs, or accession numbers that may be used to access these data. Or, if the data are not a core part of the research being presented in your study, we ask that you remove the phrase that refers to these data.**

5. Thank you for your clarification. We modified it.

**6. Please amend your list of authors on the manuscript to ensure that each author is linked to an affiliation. Authors’ affiliations should reflect the institution where the work was done (if authors moved subsequently, you can also list the new affiliation stating “current affiliation:….” as necessary).**

6. Corrected. There was a mistake in one of author affiliation (sorry).

Thank you again.

**Response to Reviewers:**

**Reviewer #1:**

**The authors report an important research regarding “Protective effect of bosentan against pulmonary hypertension development in systemic sclerosis patients with digital ulcers.”**

**The major weaknessess are lack of novelty and right heart catheterization to better define pulmonary artery hypertension.**

Thank you for your comments. It's true that the main weakness of our study was the absence of the majority of RHC to design better than using an estimated sPAP by echocardiography. But our retrospective design and to use a historical cohort (with patients followed previous to current PAH detection recommendations by RHC) limits our work in this point.

**Minor points:**

**- The authors report in the introduction “…Pulmonary Arterial Hypertension (PAH) is the most frequent cause of PH in SSc and a leading cause of death” but it is not clear in the article if they are evaluating PAH or PH. Moreover any patients showed left ventricular dysfunction, are those patients affected by PH group II? What about group III?**

- Your comment is true definitely. Some patients can present group II or III (or combined I-II / I-III) of PH in SSc. But it is know that PAH (group I) is the most important cause of PH in SSc and is critical to find or to treat in the first phases of the (PH) disease. On the other hand bosentan only demonstrated be useful to treat group I PH (PAH), and our intention was in this retrospective study to investigate if (as in DUs) it drug can prevent PAH also. But (and in association with your comment previous your minor points) we can not are sure what kind of PH is in our patients without an haemodynamic study. Taking in consideration that we used echocardiography we use PH term. But our intention was evaluate the risk of PAH. Linked with this explanation we investigated and showed in the manuscript the effect of bosentan in other risk factors associated with PAH in SSc (DLCO).

**-There were patients who were treated only with f PDE-5 inhibitor for treatment of digital ulcers?**

- Thank you for your question. A total of 31 patients were in treatment for DUs with PDE-5 inhibitors. (23 concomitantly in bosentan group and eight in non bosentan group) as we shown in table 1.

Thank you again for your constructive comments.

**Reviewer #2:**

**A retrospective study investigating the role of bosentan in the prevention of Systemic Sclerosis- Pulmonary Arterial Hypertension (SSc-PAH) is of high interest even though there are lots of bias explaining that the title should be less affirmative. There are several issues/comments:**

**Major comments:**

**1.Title:**

**This study did not demostrates the effect of bosentan in the prevention of SSc-PAH. It described the occurrence of PAH in different groups of patients taken bosentan or not. The observational nature of the study does not allow to prospect any cause/effect relationship. Well-designed randomised controlled trials (RCTs) are needed to either support or refuse this hypothesis.**

1.Thank you for your observation. we only can agree with you. We changed the title for other more appropriate with our study.

**2.Material and methods (page 5):**

**-The variable, non-standardised length of follow-up represents a limitation.**

- It’s true. We included in the limitations of our study (page 15).

**-The incidental finding of high systolic pulmonary artery pressure on echocardiography is common and is not enough to diagnose pulmonary hypertension. Conditions associated with high systolic PA pressure on echocardiogram are not necessarily associated with PAH. Have you excluded patients with hemodynamically significant valvular disease, a history of uncontrolled systemic hypertension, hyperlipidaemia, cardiac failure, hepatic failure, diabetes, cerebrovascular diseases, peripheral vascular diseases, coagulopathy, smokers and pregnant or breastfeeding women?**

- Again you are absolutely on right. We did not exclude the major part of this risk factors by missing data in reviewed database (with an important number of historical patients). We also cited the lack of some epidemiological data in the limitations of our study (pages 15 & 16). We are sorry but we can’t recover this kind of missing data. On the other hand we think that, taking into consideration all those limitations (and the difficult to obtain data about this complication in patients with a rare disease), are relevant to take into account.

**3.Statistical analysis (page 6):**

**-Please, clarify more clearly which variables are included in multivariate regression analysis.**

- We re-edited the explanation with the intention to do this part more clear (page 6).

**4.Results (page 7):**

**-Please specify in the results section why 15 of 237 enrolled patients were excluded from the study.**

- We included the explanation in Results (page 7).

**5.Table 1 (page 9):**

**- Please indicate the age, the prevalence of anticentromere and anti-topoisomerase I antibodies in both treatment and control group and the respective p-values.**

- Done.

- **Although it is known, please explain the acronym dcSSc in the table.**

- Done.

**- Please, add the p-value (i.e. 1.00) to the digital ulcers row and edit the term “Digital ulcers” to “Active digital ulcers or history of digital ulcers” as defined in Material and Methods section.**

- Done.

**- I suggest using the term Renal involvement instead of SRC.**

- Thank you for the suggestion. Done.

**- Risk factors for SSc-PAH such as ACA, older age, longer disease duration, limited cutaneous SSc subset and presence of ILD might be considered (Y. Jianga et al. Autoimmunity Rev. 2020). I suggest therefore to add disease duration to baseline characteristics.**

- Thank you for your suggestion. We included disease duration in table 1.

**- About half of the cohort (49.1%) presented ILD. I suggest to specify other treatments (i.e. corticosteroids, immunosuppressive agents, cytotoxic drugs, or antifibrotic drugs) that could affects the study results.**

- We added the immunosuppressive agents in the table 1.

**Minor comments:**

**1.Abstract (page 2 line 3):**

**- In the Introduction I suggest to add the acronym PH (third line) if you want to use it later in the objective section.**

- Thank you for you comment. We changed “PH” for pulmonary hypertension ( in order to avoid acronyms in the abstract). We also modified the abstract according style requirements of PLOS ONE.

**2.Figure 3:**

**-Please edit the IC of Prostanoids to (1,0-7,0) as reported in the text (page 11).**

- Thank you for your observation. Corrected.

**3.Discussion (page 13):**

**-Please edit Discusion in Discussion.**

- We apologize by mistake. Corrected.

Thank you again for your constructive comments and suggestions.
